# Production of Hexanol as the Main Product Through Syngas Fermentation by *Clostridium carboxidivorans* P7

**DOI:** 10.3389/fbioe.2022.850370

**Published:** 2022-04-25

**Authors:** Hyun Ju Oh, Ja Kyong Ko, Gyeongtaek Gong, Sun-Mi Lee, Youngsoon Um

**Affiliations:** ^1^ Clean Energy Research Center, Korea Institute of Science and Technology, Seoul, South Korea; ^2^ Division of Energy and Environment Technology, KIST School, University of Science and Technology (UST), Daejeon, South Korea

**Keywords:** *Clostridium carboxidivorans*, hexanol, syngas (CO/CO2/H2), acetogen, ethanol

## Abstract

The production of hexanol from syngas by acetogens has gained attention as a replacement for petroleum-derived hexanol, which is widely used in the chemical synthesis and plastic industries. However, acetogenic bacteria generally produce C2 compounds (e.g., acetate and ethanol) as the main products. In this study, the gas fermentation conditions favorable for hexanol production were investigated at different temperatures (30–37°C) and CO gas contents (30–70%) in batch gas fermentation. Hexanol production increased from 0.02 to 0.09 g/L when the cultivation temperature was lowered from 37 to 30°C. As the CO content increased from 30 to 70%, the CO consumption rate and hexanol production (yield, titer, and ratio of C6 compound to total products) increased with the CO content. When 70% CO gas was repeatedly provided by flushing the headspace of the bottles at 30°C, the total alcohol production increased to 4.32 g/L at the expense of acids. Notably, hexanol production (1.90 g/L) was higher than that of ethanol (1.20 g/L) and butanol (1.20 g/L); this is the highest level of hexanol produced in gas fermentation to date and the first report of hexanol as the main product. Hexanol production was further enhanced to 2.34 g/L when 2 g/L ethanol was supplemented at the beginning of 70% CO gas refeeding fermentation. Particularly, hexanol productivity was significantly enhanced to 0.18 g/L/day while the supplemented ethanol was consumed, indicating that the conversion of ethanol to acetyl-CoA and reducing equivalents positively affected hexanol production. These optimized culture conditions (gas fermentation at 30°C and refeeding with 70% CO gas) and ethanol supplementation provide an effective and sustainable approach for bio-hexanol production.

## Introduction

Renewable energy resources are required to cope with oil resource depletion and climate change caused by carbon dioxide emissions generated during petrochemical production. The use of microbes to produce biofuels and biochemicals from renewable resources has gained attention because of the sustainability of these process and concerns related to global climate change (Clomburg et al., 2017). Biomass used in bio-manufacturing has expanded from food biomass (e.g., corn) to lignocellulosic biomass and algae (Jang et al., 2012). However, the cost of raw materials accounts for over 50% of the total cost of biofuel/chemical production, and certain components in biomass (e.g., lignin and non-fermentable sugars) remain as waste, resulting in incomplete utilization of carbon in the biomass (Lee et al., 2016; Moon et al., 2016). Alternatively, syngas (a gaseous mixture of CO, CO_2_, and H_2_) has recently attracted attention as an economical carbon source for microorganisms because syngas is evolved as waste gas from the steel-manufacturing process and syngas fermentation is one way to utilize whole lignocellulosic biomass, including lignin, without fractionation and saccharification processes (Lagoa-Costa et al., 2017; Im et al., 2019; Thi et al., 2020). Hence, the production of biofuels and chemicals from syngas can secure economic feasibility and reduce carbon dioxide emission (Sun et al., 2019).

Acetogens can utilize syngas through metabolic circuits that synthesize acetate and other metabolites (Dürre and Eikmanns, 2015). In many recent studies, acids and solvents were produced from syngas using acetogens, which has the advantages of a low substrate cost, abundance of raw materials, and high carbon recovery yield (Jones et al., 2016; Marcellin et al., 2016). Acetogens produce acetyl-CoA from syngas via the Wood–Ljungdahl pathway. Representative acetogens include *Acetobacterium woodii* (Weghoff et al., 2015), *Clostridium aceticum* (Poehlein et al., 2015), *Clostridium autoethanogenum* (Im et al., 2021), and *Clostridium ljungdahlii* (Köpke et al., 2010). Although acetogen strains mainly produce C2 compounds (acetate and ethanol) from syngas, they are also known to produce C4–C6 acids and alcohols (Charubin et al., 2018; Bao et al., 2019; Im et al., 2021). Among them, *C. carboxidivorans* P7 (Phillips et al., 2015) and *C. ragsdalei* (Ramachandriya et al., 2013) were reported to produce 0.94 and 0.09 g/L of hexanol, respectively, from syngas.

Biosynthesized hexanol can be used as a renewable C6 platform material and an alternative biofuel to conventional transportation fuels (De Poures et al., 2017). Bio-hexanol is a long-chain bio-alcohol compared to intensively researched biofuels such as bioethanol and bio-butanol. Compared to ethanol and butanol, hexanol shows a higher cetane index (8, 17, and 23 for ethanol, butanol, and hexanol, respectively) and energy density (26.83, 33.09, and 39.10 MJ/kg for ethanol, butanol, and hexanol, respectively) (De Poures et al., 2017). In addition, hexanol can be chemically converted into 1-hexene, a platform substance used in various petrochemical products (Harvey and Meylemans, 2014).

Although there has been great interest in producing hexanol from syngas using *C. carboxidivorans* P7, the titer of hexanol was generally <1 g/L (Phillips et al., 2015). Shen et al. (2017) performed time-controlled fermentation at two different temperatures (37°C for 0–24 h and 25°C for 24–144 h) and achieved a total alcohol titer of 6.97 g/L, including 1.33 g/L hexanol. However, the ethanol titer (3.97 g/L) was much higher than that of hexanol. Previous studies showed that *C. carboxidivorans* P7 tends to produce acids or alcohols depending on the culture conditions and fermentation techniques used (Phillips et al., 2015; Ramió-Pujol et al., 2015). To produce hexanol as the main product with a higher titer, culture conditions favorable for chain elongation to C6 as well as alcohol production should be identified. Therefore, this study was conducted to improve hexanol production by optimizing the cultivation temperature and CO gas content for gas fermentation of *C. carboxidivorans* P7. The effects of refeeding CO-rich gas and ethanol supplementation on hexanol production were investigated, and hexanol production was enhanced to the highest level reported to date.

## Materials and Methods

### Microorganism, Medium, and Culture Conditions

*Clostridium carboxidivorans* P7 (DSMZ 15243) was obtained from the German Collection of Microorganisms and Cell Cultures (Braunschweig, Germany). 2xYTG (Herman et al., 2017) medium was used to grow the seed culture of *C. carboxidivorans* P7 for use in fermentation. Bacterial glycerol stocks were maintained at −80°C and inoculated (5% [v/v]) into 57 ml serum bottles containing 20 ml 2xYTG medium comprised of (per liter of distilled water) 5 g of glucose, 16 g of tryptone, 10 g of yeast extract, 0.9 g of sodium chloride, and 0.001 g of resazurin. The 2xYTG medium was purged using Ar gas to maintain anaerobic conditions and then autoclaved at 121°C at 1.2 atm for 20 min. The seed culture was incubated at 30°C in an incubator shaker set at 150 rpm.

When the cells reached an optical density (OD) of 2–2.5 at a wavelength of 600 nm (mid-exponential phase), the cultured bacterial cells were transferred (5% [v/v]) to modified P7-S medium (MP7-S) for gas fermentation adapted from Phillips et al. (2015). The MP7-S medium consisted of (per liter of distilled water) 2 g of yeast extract, 2 g of NH_4_Cl, 0.08 g of CaCl_2_·2H_2_O, 0.4 g of MgSO_4_·7H_2_O, 0.2 g of KCl, 0.2 g of KH_2_PO_4_, 0.001 g of resazurin, 0.2 g of HCl-l-cysteine, and 1 ml of additional trace elements. Trace elements contained (per liter of distilled water) 20 mg of nitrilotriacetic acid, 20 mg of MnSO_4_·H_2_O, 8 mg of Fe(SO_4_)_2_(NH_4_)_2_·6H_2_O, 2 mg of CoCl_2_·6H_2_O, 0.002 mg of ZnSO_4_·7H_2_O, 0.2 mg of CuCl_2_·2H_2_O, 0.2 mg of NiCl_2_·2H_2_O, 2.2 mg of NaMoO_4_·2H_2_O, 0.2 mg of Na_2_WO_4_, 0.2 mg of KAl(SO_4_)_2_·12H_2_O, and 0.1 mg of H_3_BO_3_. For pH buffering of the batch cultures in serum bottles, 50 mM 2-(N-morpholino) ethanesulfonic acid hydrate (MES) was added, and the initial pH of the medium was adjusted to 6 with 2 M KOH. The MP7-S medium was purged with Ar gas to maintain anaerobic conditions and sterilized at 121°C for 20 min. The cells were cultivated at 30°C with shaking at 150 rpm. A total of 150 kPa of the gas mixture was pressurized into the headspace of the serum bottles (20 ml of MP7-S medium in a 157 ml serum bottle).

### Fermentation

Batch fermentation was performed in 157 ml serum bottles containing 20 ml MP7-S medium supplemented with syngas at 1.5 bar. A syngas composition of CO:CO_2_:H_2_ = 30:30:40 was used to investigate the effect of temperature on gas fermentation. The bottles were incubated at 30, 33, and 37°C. After selecting 30°C as the optimum temperature, gas fermentation was continued at this temperature. In experiments using different CO gas contents, gas mixtures with CO:Ar ratios of 30:70, 50:50, and 70:30 were supplied. To investigate the effects of adding acetate and ethanol separately or together, acetate (in the form of CH_3_COONa) and ethanol were added to the MP7-S medium at a concentration of 2 g/L; a CO:Ar ratio of 70:30 was used. For gas-refeeding fermentation, the headspace was filled with 150 kPa of CO:Ar = 70:30. A mixture of CO:Ar = 70:30 was refed every 72 h. All experiments were performed in duplicate.

### Analytical Methods

Cell growth was evaluated by measuring the OD at 600 nm using a UV-visible spectrophotometer (Cary 60, Agilent Technologies, Santa Clara, CA, United States). The pH was measured using a pH meter (Thermo Fisher Scientific, Waltham, MA, United States). The compositions of H_2_, CO, and CO_2_ were analyzed using a gas chromatograph (GC) (6890N, Agilent Technologies) equipped with a thermal conductivity detector. The gas partial pressure was calculated using the calibration curve (mole vs GC-thermal conductivity detector peak area) and ideal gas equation. For example, 5.4 mmol CO in the headspace (with 137 ml headspace in 157 ml serum bottle at 30°C) was equivalent to 100 kPa of CO partial pressure. Metabolites (ethanol, butanol, hexanol, acetate, butyrate, and hexanoate) were analyzed using a GC equipped with a flame ionization detector and 30 m × 0.32 mm × 0.25 μm HP-INNOWAX polyethylene glycol column (Agilent Technologies). Helium was used as the carrier gas. The temperature of the oven was increased from 50 to 170°C at a rate of 10°C/min, and the injector and detector temperatures were both set to 250°C. Before GC analysis, the sample was centrifuged, and the supernatant was filtered through a 0.2 μm pore size syringe filter (Oh et al., 2019).

## Results and Discussion

### Effect of Cultivation Temperature on Hexanol Production

**FIGURE 1:**
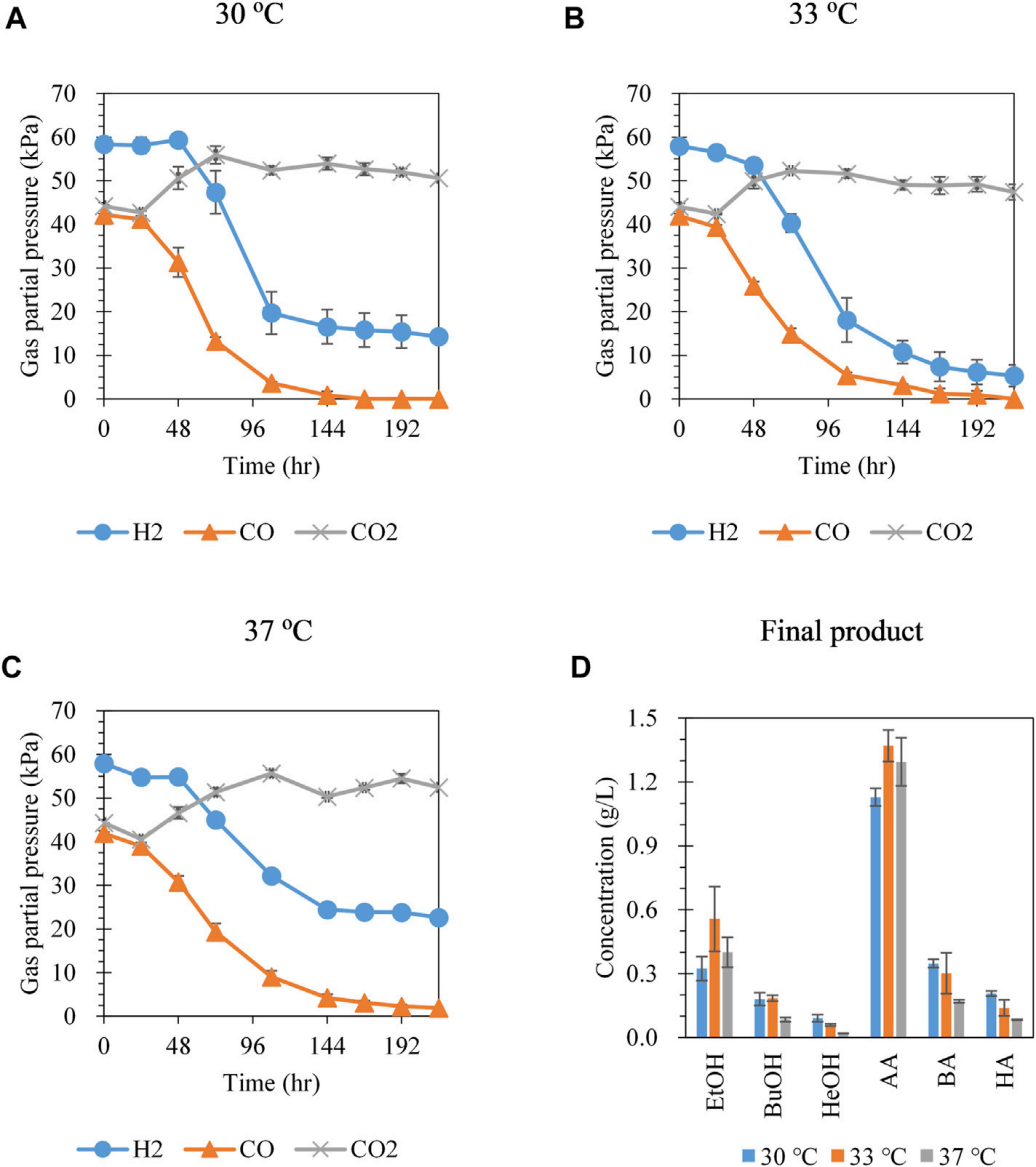
Gas pressure profiles at **(A)** 30°C, **(B)** 33°C, and **(C)** 37°C and **(D)** metabolite concentration at 216 h from syngas fermentation by *C. carboxidivorans* P7. The headspace was filled with syngas CO:CO_2_:H_2_ = 30:30:40 at an initial headspace pressure of 150 kPa. Each data point and error bar represent the average and range of duplicate experiments, respectively. EtOH, ethanol; BuOH, butanol; HeOH, hexanol; AA, acetic acid; BA, butyric acid; HA, hexanoic acid.

Hexanol production was investigated at different cultivation temperatures (30, 33, and 37°C) with syngas (CO:CO_2_:H_2_ = 30:30:40) at an initial headspace pressure of 150 kPa. As the cultivation temperature was increased, the CO consumption rate decreased. CO was completely consumed after 168 and 216 h at 30 and 33°C, respectively (Figures 1A,B); however, CO remained until the end of cultivation at 37°C (1.9 kPa at 216 h) (Figure 1C). H_2_ remained for 216 h at all temperatures, with the largest amount of H_2_ consumed at 33°C, followed by at 30 and 37°C. Hexanol production increased with decreasing cultivation temperatures, showing values of 0.09, 0.06, and 0.02 g/L at 30, 33, and 37°C, respectively (Figure 1D). Although the total consumption of CO and H_2_ at 33°C was greater than that at 30°C, the production of hexanol at 33°C was lower than that at 30°C. Interestingly, the ratio of C6 compounds among the total C2–C6 compounds produced (i.e., total mass of C6 compounds divided by the total mass of C2–C6 compounds) also increased with decreasing cultivation temperatures (13.1, 7.59, and 4.99 wt % at 30, 33, and 37°C, respectively) (Supplementary Figure S1). This result suggests that the tendency for chain elongation was greater at 30°C than at higher temperatures. Cell growth (OD at 600 nm) increased with decreasing cultivation temperatures (Supplementary Figure S1). Based on the results shown in Figure 1, subsequent experiments for hexanol production were conducted at 30°C.

Shen et al. (2020) demonstrated that the production of higher alcohols was enhanced at 25°C compared to that at 37°C, although the optimal temperature for *C. carboxidivorans* P7 growth is 37–40°C (Fernández-Naveira et al., 2017). It was reported that genes related to the Wood-Ljungdahl pathway were highly expressed at 37°C, whereas alcohol synthesis-related genes were relatively highly expressed at 25°C (Shen et al., 2020). Although several previous studies demonstrated that the production of long-chain alcohols was improved at low temperatures, the main product was ethanol, and the hexanol concentration was much lower than that of ethanol (Ramió-Pujol et al., 2015; Zhang et al., 2016; Shen et al., 2017; Shen et al., 2020). Therefore, further optimization of the gas fermentation process is required to produce hexanol as the main product.

### Effect of CO Gas Pressure on Cell Growth and Hexanol Production

Although CO gas is an energy and carbon source for acetogens, it also exhibits a substrate-inhibitory effect at high CO pressures (Ramió-Pujol et al., 2015; Lanzillo et al., 2020). Therefore, an adequate supply of CO gas is necessary to stimulate CO consumption with a short lag phase. To investigate the inhibitory effect of CO on *C. carboxidivorans* P7, different compositions of gas mixtures with CO:Ar ratios of 30:70, 50:50, and 70:30 were used at an initial total pressure of 150 kPa and 30°C. In the early stage of fermentation (up to 48 h), the rate of CO consumption increased with an increasing CO supply, with the highest consumption rate of 0.38 kPa/h at 70% CO (Table 1) without a prolonged lag phase in cell growth (Supplementary Figure S2). As the CO supply increased from 30 to 70%, the hexanol titer and yield also increased (Table 1), indicating an increase in the metabolic flux for hexanol synthesis under 70% CO conditions. The partial pressure of CO also affected the ratio of chain elongation to C6 compounds (hexanoate and hexanol) among all C2–C6 metabolites: the ratio of C6 compounds increased with increasing CO partial pressures up to 26.7 wt % when 70% CO was supplied (Table 1).

**TABLE 1:** Effect of CO ratio on fermentation performance with the initial headspace pressure of 150 kPa.

Initial CO ratio (CO:Ar)	30:70	50:50	70:30
CO consumption rate (kPa/h) during 48 h	0.34 ± 0.02	0.37 ± 0.02	0.38 ± 0.01
Hexanol titer (g/L)	0.03 ± 0.00	0.05 ± 0.00	0.10 ± 0.01
Hexanol yield (hexanol g/CO g)	0.0092 ± 0.0004	0.0094 ± 0.0005	0.0137 ± 0.0022
C6 ratio (wt %)^a^	13.2 ± 0.02	18.9 ± 0.02	26.7 ± 0.03

Values are the average and range of duplicate experiments.

a C6 ratio (wt %) = [(hexanoate + hexanol) g]/(total product g) × 100%.

Lanzillo et al. (2020) reported the effect of the partial pressure of CO on the growth of *C. carboxidivorans* P7 and metabolite production with different liquid-to-gas volume ratios (V_L_/V_G_). According to this report, cell growth was not inhibited at a partial pressure of CO below 1.1 atm regardless of the tested V_L_/V_G_ (0.28 and 0.92). In this study, as shown in Supplementary Figure S2, *C. carboxidivorans* P7 consumed CO without being hindered by 70% CO in the early stages of fermentation at a V_L_/V_G_ ratio of 0.15. The initial partial pressure of CO was approximately 0.95 atm, which agreed with the results of Lanzillo et al. (2020) regarding the inhibitory effect of CO. Hexanol production increased with the partial pressure of CO in this study; Lanzillo et al. (2020) did not report hexanol production because of the negligible amounts produced. A possible reason for the different hexanol production patterns is the different cultivation temperatures and V_L_/V_G_ between our study and that by Lanzillo et al. (2020). The cultivation temperatures were 30 and 35°C in this study and a study conducted by Lanzillo et al. (2020), respectively. According to Figure 1 and previous reports (Ramió-Pujol et al., 2015; Zhang et al., 2016; Shen et al., 2017; Shen et al., 2020), a low temperature of 30°C appeared to positively affect hexanol production. In addition, considering that a sufficient CO supply is essential for chain elongation and alcohol production as a reducing equivalent and carbon source, the lower ratio of V_L_/V_G_ in this study (0.15 at an initial CO pressure of 0.95 atm at 30°C) compared to that used by Lanzillo et al. (2020) (0.28 and 0.92 at an initial CO pressure of 0.5–2.5 atm at 35°C) may enable the production of hexanol by *C. carboxidivorans* P7.

The hexanol production and yield increased with increasing partial pressures of CO (Table 1). The increase in CO pressure improves the solubility and mass transfer of CO in water, increasing the availability of CO for *C. carboxidivorans* P7 as a carbon source and reducing the equivalent source. According to Lanzillo et al. (2020), when the partial pressure of CO was increased from 0.5 to 1.5 atm at a V_L_/V_G_ of 0.28, ethanol, butanol, and hexanoate increased by 2.4-, 1.7-, and 1.7-fold, respectively. The production of acetate and butyrate did not increase with increases in the partial pressure of CO. At a V_L_/V_G_ of 0.92, the effect of the partial pressure of CO on metabolite production was less significant than that of a V_L_/V_G_ of 0.28.The production of butyrate and butanol increased with the CO partial pressure (from 0.5 to 1.5 atm) by 1.3- and 1.4- fold, respectively, but there was no increase in other metabolites. Considering the effect of CO pressure at a V_L_/V_G_ of 0.28 and 0.92, 1) high CO partial pressures (regardless of V_L_/V_G_ ratio) may have promoted the production of alcohols and high carbon chain products, which are metabolites with a high reducing equivalent demand, and 2) the effect of CO pressure on chain elongation and alcohol production may have been more pronounced at a lower liquid-to-gas volume ratio. As the ratio of V_L_/V_G_ was 0.15 in this study which was much lower than the V_L_/V_G_ of 0.28 tested by Lanzillo et al. (2020), the effect of CO partial pressure on chain elongation and alcohol production was likely more significant than that reported by Lanzillo et al. (2020), resulting in an increase in hexanol production and yield. Overall, gas fermentation with a low V_L_/V_G_ ratio (0.15) and high CO pressure (70% CO) at a low temperature (30°C) appeared to enhance hexanol production.

### Effect of Supplemented C2 Compounds on Hexanol Production

**FIGURE 2:**
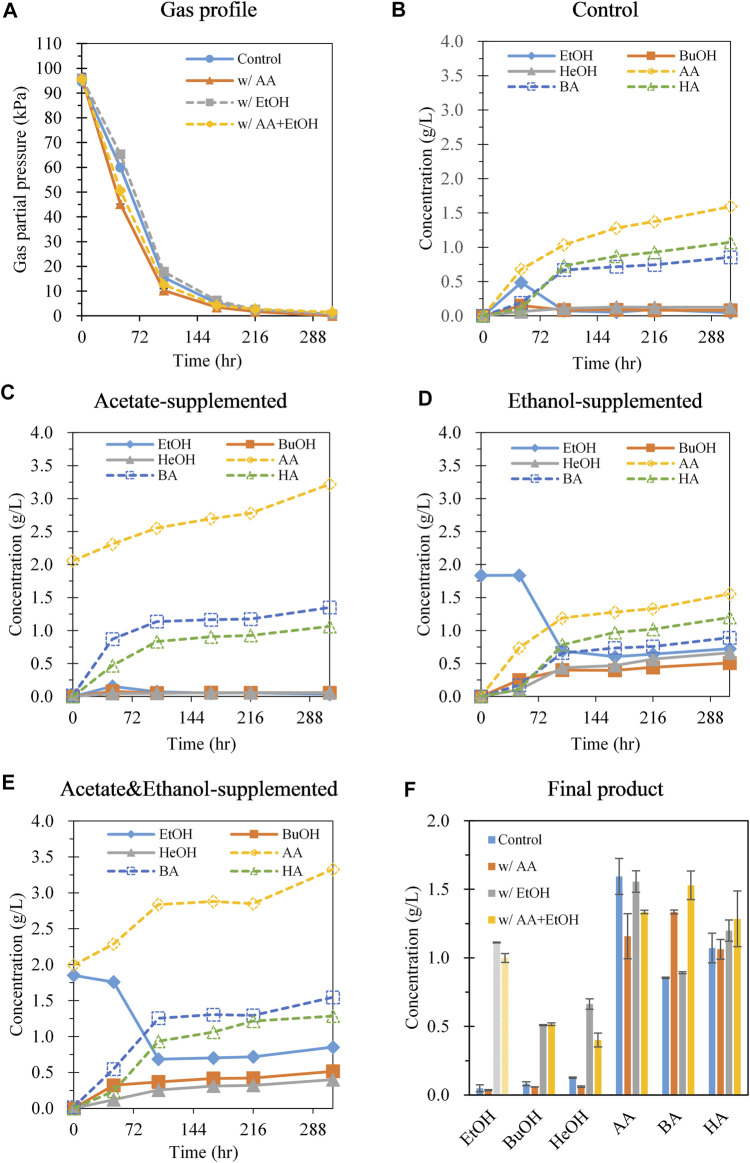
Effect of adding C2 compounds (acetate, ethanol, and acetate + ethanol) on gas consumption and metabolite production during batch gas fermentation with 70% CO gas. **(A)** Gas pressure profiles; **(B)** control (no addition of acetate and ethanol); **(C)** addition of acetate (2 g/L); **(D)** addition of ethanol (2 g/L); **(E)** addition of acetate (2 g/L) and ethanol (2 g/L); **(F)** metabolite concentration at 312 h. Shade pattern represents ethanol consumption. Each data point and error bar represent the average and range of duplicate experiments, respectively. EtOH, ethanol; BuOH, butanol; HeOH, hexanol; AA, acetic acid; BA, butyric acid; HA, hexanoic acid.

Although cultivation of *C. carboxidivorans* P7 at 30°C with 70% CO led to increased hexanol production, the main product was acetate, and chain elongation up to C6 compounds was limited. Acetogens can utilize the Wood–Ljungdahl pathway to synthesize acetyl-CoA, which can be converted to butyryl-CoA and further to hexanoyl-CoA using reducing equivalents (e.g., NADH) via chain elongation metabolic pathways (Fernández-Naveira et al., 2017). Each acyl-CoA molecule can be converted to its corresponding acid and alcohol. As acetyl-CoA is a common intermediate in the synthesis of C2, C4, and C6 compounds, increasing the acetyl-CoA flux towards chain elongation rather than the synthesis of C2 compounds (acetate and ethanol) should be considered. To reduce the production of C2 compounds, we supplemented acetate and ethanol separately or together to decrease the conversion of acetyl-CoA to acetate or ethanol through product inhibition. In this case, acetyl-CoA generated from the Wood-Ljungdahl pathway is expected to proceed to the chain elongation pathway. Meanwhile, it was found that ethanol was produced at an early stage of gas fermentation and then decreased as fermentation progressed (Figures 2A,B). This implies that the produced ethanol may be converted to acetyl-CoA with the generation of reducing equivalents. Because acetyl-CoA and reducing equivalents are in high demand for both chain elongation and alcohol production (Fernández-Naveira et al., 2017), ethanol supplementation is expected to influence the metabolite profiles.

When *C. carboxidivorans* P7 was fermented in the presence of 2 g/L acetate under a CO:Ar ratio of 70:30 at 150 kPa, the rate of CO consumption increased during the first 48 h (1.06 kPa/h) compared with that in the control without acetate addition (0.72 kPa/h) (Figure 2A). At the end of fermentation, net acetate production (subtracting the initial concentration from the final value) with acetate supplementation was 1.16 g/L, whereas acetate production in the control culture was 1.59 g/L. This result indicates that acetate supplementation effectively decreased acetate production. Ethanol was produced in small amounts with acetate supplementation, whereas ethanol production followed by ethanol reassimilation was observed in the control culture (Figure 2B). Interestingly, butyrate production increased when acetate was added (1.33 g/L with acetate vs 0.85 g/L without acetate), but there was no change in the hexanoate levels, and hexanol production was decreased (0.06 g/L with acetate vs 0.13 g/g without acetate) (Figures 2C,F). The ratio of C6 to C4 compounds (g/g) with acetate supplementation was 0.81, whereas this ratio was 1.28 without acetate. This change in the metabolite distribution suggests that the supplemented acetate influenced acetyl-CoA flux toward chain elongation to butyrate, but not to hexanoate and hexanol. The ratio of C4–C6 alcohol/C4–C6 acids was 0.05 and 0.11 with and without acetate, respectively, illustrating that the tendency to produce alcohol decreased when acetate was supplemented (Supplementary Table S1). The pH values were higher in the sample with acetate supplementation after 96 h because of the buffering action of the supplemented acetate (Supplementary Figure S3).

When 2 g/L ethanol was added, the rate of CO consumption was similar to that in the control (Figure 2A). Notably, the concentration of ethanol dramatically decreased to 0.69 g/L during 102 h of cultivation, and then slightly increased after 168 h of cultivation (Figure 2D). The overall ethanol consumption was 1.11 g/L (Figure 2F). Comparison of the metabolite profiles with and without ethanol showed that acetate and butyrate production did not show large changes. In contrast, butanol production significantly increased by 6.18-fold (0.51 g/L with ethanol vs 0.08 g/L without ethanol), and hexanol production showed a considerable 5.26-fold increase (0.66 g/L with ethanol vs 0.13 g/L without ethanol) (Figures 2D,F). During 102 h of cultivation during which the ethanol concentration decreased, ethanol consumption was 1.15 g/L (0.49 mmol) and hexanol production was 0.44 g/L (0.086 mmol). As CO consumption (4.29 mmol) was similar regardless of ethanol supplementation, the difference in hexanol production with and without ethanol supplementation during 102 h of cultivation (0.44 vs 0.11 g/L) was likely caused by ethanol consumption. Therefore, the yields of hexanol derived from ethanol and CO gas were estimated as 0.13 mole hexanol/mole ethanol and 0.0051 mole hexanol/mole CO, respectively. Similarly, the yields of butanol derived from ethanol and CO gas were 0.17 mol butanol/mole ethanol and 0.0054 mol butanol/mole CO, respectively, demonstrating that ethanol supplementation enhanced the production of both hexanol and butanol. The theoretical amount of ethanol required to increase butanol and hexanol production was 0.36 mmol (refer to Supplementary Figure S4 for theoretical stoichiometry equations). Thus, approximately 73% of the consumed ethanol (0.36 mmol/0.49 mmol) appeared to contribute to improving butanol and hexanol production. At the end of fermentation, the ratio of C6 to C4 compounds (g/g) with ethanol supplementation was 1.33, which was similar to that without ethanol supplementation (1.28). The ratio of C4–C6 alcohols to C4–C6 acids was 0.56, which was much higher than that without ethanol (0.11), suggesting that C4–C6 alcohol production was facilitated by utilizing the supplemented ethanol (Supplementary Table S1). The pH drop with ethanol supplementation after 96 h was less than that without ethanol supplementation (Supplementary Figure S3), likely because of the higher alcohol production compared to in the control.

When acetate and ethanol were supplemented together, the rate of gas uptake increased (0.94 kPa/h) over 48 h. The net acetate production decreased (1.33 g/L) and butyrate production increased (1.53 g/L) (Figure 2E) compared to that in the control (Figure 2B). This result was similar to that observed in cultures with acetate (Figure 2C). The ethanol concentration decreased to 0.69 g/L during 48–102 h of cultivation; additionally, butanol (0.52 g/L) and hexanol production increased (0.40 g/L) compared to in the control (Figures 2E,F). The ratio of C6 to C4 compounds (g/g) with simultaneous supplementation of acetate and ethanol was 0.82, which was similar to the ratio obtained with acetate supplementation alone (0.81) but lower than that with ethanol supplementation alone (1.33) (Supplementary Table S1). The ratio of C4–C6 alcohols to C4–C6 acids was 0.33, which is the median value of the ratios between acetate supplementation (0.11) and ethanol supplementation (0.56). Therefore, no synergistic effect was observed with both acetate and ethanol supplementation with respect to chain elongation to C6 compounds (Supplementary Table S1).

Zhang et al. (2016) examined the gas fermentation profiles of *C. carboxidivorans* P7 with supplemented end products, such as acetate (2–8 g/L), butyrate (2–8 g/L), hexanoate (0.5–3 g/L), ethanol (4–16 g/L), and butanol (4–16 g/L) at both 25 and 37°C. Zhang et al. (2016) determined the cell biomass-specific yield of the end product (g/g, production of end product normalized to maximum cell biomass); thus, the concentrations of end products between this study and the study conducted by Zhang et al. (2016) could not be directly compared. Comparison of metabolite production following acetate supplementation revealed a decreased ratio of hexanoate/butyrate in both our study and that by Zhang et al. (2016); however, the enhanced ethanol production observed by Zhang et al. (2016) was not observed in our study. When ethanol was supplied, an increase in the ratio of butanol/hexanol production was observed in both studies, whereas the increase in acetate production described by Zhang et al. (2016) was not detected in this study. Based on this study and that by Zhang et al. (2016), acetate or ethanol supplementation facilitated chain elongation of C4 compounds. Zhang et al. (2016) did not examine the effect of acetate and ethanol supplementation on hexanol production in detail, whereas we observed that ethanol consumption notably increased butanol and hexanol production compared with that in the control. Solvent production by clostridia requires sufficient reducing equivalents; ethanol was likely metabolized and used as an electron and acetyl-CoA donor, leading to increased hexanol production by *C. carboxidivorans* P7 (Supplementary Figure S4).

### Effect of CO Gas Refeeding and Supplementing Ethanol on Hexanol Production

**FIGURE 3:**
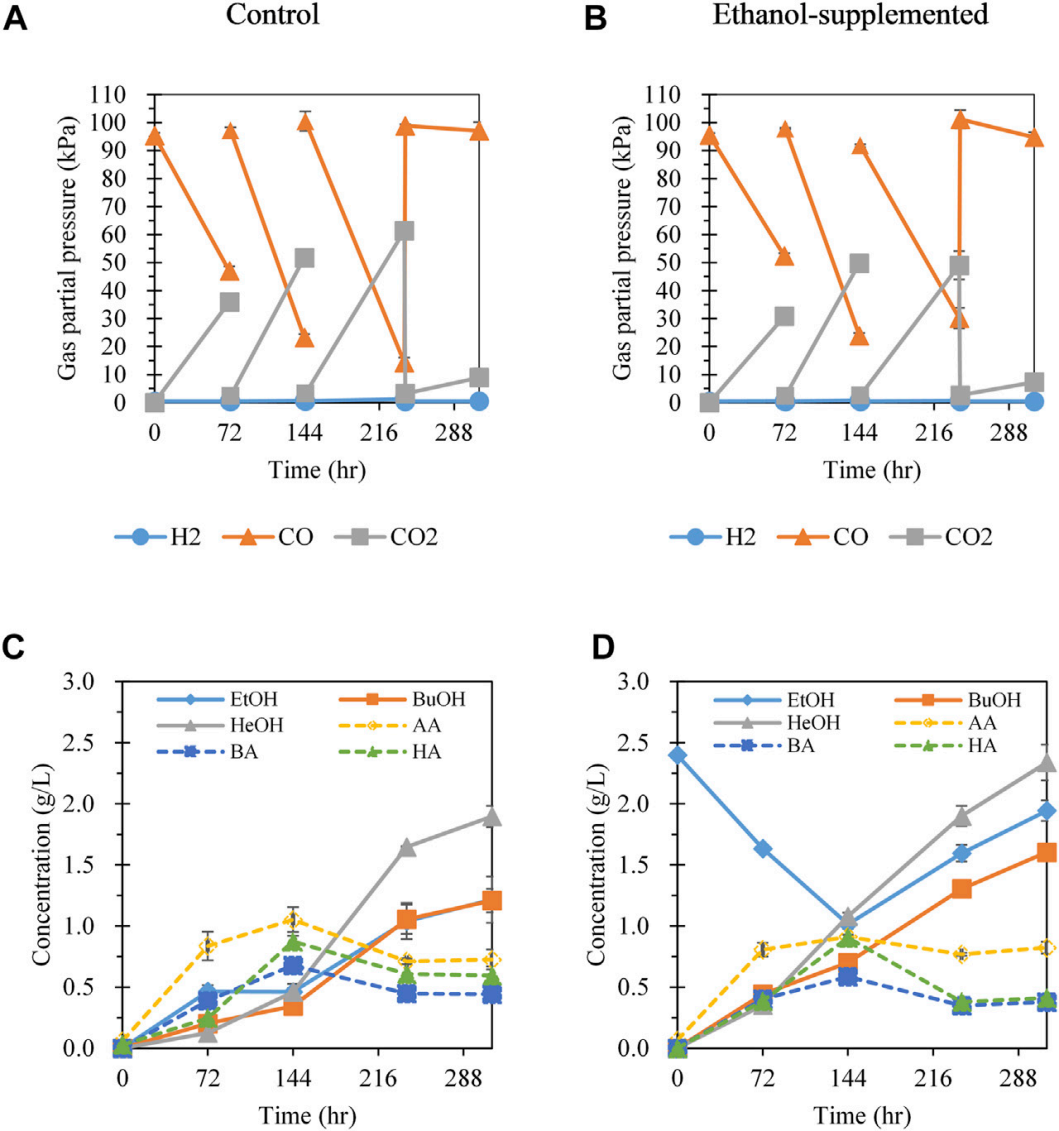
Effect of 70% CO gas refeeding and addition of ethanol on gas consumption and metabolite production. **(A)** Gas pressure profiles with CO gas refeeding at 72, 144, and 240 h; **(B)** gas pressure profiles with initial addition of ethanol (2 g/L) and CO gas refeeding at 72, 144, and 240 h; **(C)** product profiles with gas refeeding; **(D)** products profiles with ethanol addition and gas refeeding. Each data point and error bar represents the average and range of duplicate experiments, respectively. EtOH, ethanol; BuOH, butanol; HeOH, hexanol; AA, acetic acid; BA, butyric acid; HA, hexanoic acid.

According to the results of batch gas fermentation, the cultivation of *C. carboxidivorans* P7 at 30°C with CO:Ar = 70:30 gas and ethanol supplementation was favorable for hexanol production. To further enhance hexanol production, CO gas was refed by flushing the headspace of the bottles with CO:Ar = 70:30 gas every 3 days (Figures 3A,B). CO gas refeeding fermentation was conducted with and without ethanol supplementation to investigate the effect of ethanol on CO gas refeeding fermentation.

In the absence of ethanol supplementation, acetate, butyrate, and ethanol were produced during the first 72 h of cultivation (Figures 3A,C). After refeeding of CO gas at 72 h, the hexanoate concentration significantly increased and showed the highest productivity among the metabolites (0.21 g/L/day), followed by hexanol (0.11 g/L/day) and butyrate (0.096 g/L/day). This result was accomplished by supplying sufficient CO gas, which served as an electron donor and carbon source for chain elongation up to C6 compounds. Interestingly, ethanol reassimilation (seen in Figure 2B) was not observed in the CO refeeding fermentation system. During 144–241 h of cultivation after the second CO refeeding, the concentrations of C2–C6 acids decreased, and C2–C6 alcohol production increased (Figure 3C). Hexanol was produced with the highest productivity (0.30 g/L/day), followed by butanol (0.18 g/L/day) and ethanol (0.14 g/L/day). Considering that the increase in the alcohol concentration was greater than the decrease in corresponding acid concentration during 144–241 h (increase of 11.3, 7.72, and 6.50 mM of hexanol, butanol, and ethanol, respectively; decrease of 3.26, 2.56, and 4.08 mM of hexanoate, butyrate, and acetate, respectively), alcohol production increased not only because acid was converted to alcohol but also via direct alcohol production through a metabolic shift from acidogenesis to solventogenesis. The pH increase after 144 h also supported the transition from acidogenic to solventogenic metabolism, which is generally observed in solvent-producing clostridial cultures (Han et al., 2020) (Supplementary Figure S5). After the third CO refeeding at 241 h, a small amount of CO was consumed. At the end of fermentation, total alcohol production (4.32 g/L) was much higher than total acid production (1.76 g/L), with ethanol, butanol, and hexanol concentrations of 1.21, 1.21, and 1.90 g/L, respectively. To the best of our knowledge, this is the highest hexanol production observed to date using *C. carboxidivorans* P7 from CO as the sole carbon source. Moreover, compared to previous studies in which ethanol was the main alcohol among C2–C6 alcohols, we showed that hexanol was the main product and was present at a higher concentration than that of ethanol. The ratio of C6 compounds to all products was increased from 31.7% in batch gas fermentation to 43.9% in CO gas refeeding fermentation. Additionally, the ratio of total alcohols to total acids (g/g) was also dramatically increased from 0.07 (without gas refeeding) to 2.45 with gas refeeding. Therefore, a sufficient supply of CO gas via refeeding is important for chain elongation and alcohol production.

To investigate the effect of ethanol supplementation on the fermentation performance with CO gas refeeding, 2 g/L of ethanol was added. As shown in Figures 3B,D, acetate was the main product, and ethanol was consumed during 0–72 h of cultivation, as observed in batch fermentation without CO refeeding (Figure 2D). From 72 h of cultivation after CO refeeding, the ethanol concentration decreased continuously and hexanol production increased, showing the highest productivity (0.24 g/L/day) followed by hexanoate (0.17 g/L/day) and butanol (0.086 g/L/day). Comparison of CO refeeding fermentation with and without ethanol supplementation (Figures 3B,D) revealed that C4–C6 alcohol production appeared to be promoted as supplemented ethanol was consumed during the 144 h of cultivation. The decrease in pH following ethanol supplementation over 72–144 h was less than that without ethanol supplementation, likely because of the increased alcohol production (Supplementary Figure S5). During 144 h of cultivation, the ethanol consumption was 1.38 g/L (0.60 mmol) and CO consumption was 6.37 mmol. The butanol and hexanol concentrations at 144 h were higher than those in the absence of ethanol supplementation by 0.36 g/L (0. 70 vs 0. 34 g/L) and 0.62 g/L (1.08 vs 0.46 g/L), respectively. The effect of ethanol supplementation on hexanol production was greater than that of butanol production for some reason. Further study are needed to elucidate the metabolic regulation that influences the production of butanol and hexanol upon ethanol supplementation. Assuming that ethanol consumption led to an increase in butanol and hexanol production, the yield of butanol and hexanol derived from ethanol was estimated as 0.16 mol butanol/mol ethanol and 0.20 mol hexanol/mol ethanol, respectively. The yields of butanol and hexanol derived from CO were 0.0140 and 0.0136 mol butanol/mol CO, respectively. As the theoretical amount of ethanol required to increase butanol and hexanol production was 0.56 mmol, approximately 93% of the consumed ethanol likely contributed to improving butanol and hexanol production.

When CO gas was refed at 144 h, the trends in metabolite production and pH profiles were similar to those without ethanol supplementation in terms of a decrease in C2–C6 acid concentrations, an increase in C2–C6 alcohol production, and an increase in pH (i.e., transition from acidogenesis to solventogenesis) (Supplementary Figure S5). As observed in the absence of ethanol supplementation, the increase in alcohol concentration was greater than the decrease in the corresponding acid concentration, indicating that alcohol production and the conversion of acid to alcohol occurred simultaneously. Regarding CO gas consumption after 144 h of cultivation, the consumption rate of CO with ethanol supplementation (0.65 kPa/h) was only 71% of that without ethanol supplementation (0.91 kPa/h), possibly because of the inhibitory effect of the products, such as butanol, hexanoate, and hexanol. This inhibitory effect was much higher with following ethanol supplementation than in samples without ethanol supplementation. Overall, the final concentrations of butanol and hexanol after 312 h of fermentation were 1.60 and 2.34 g/L, respectively, which were higher than those without ethanol supplementation. The ratio of C6 to C4 compounds (g/g) with ethanol supplementation was 1.39, which was slightly lower than that without ethanol supplementation (1.51). However, the C4–C6 alcohols to C4–C6 acid ratio was 4.99, which was much higher than that without ethanol (2.99), demonstrating that C4–C6 alcohol production was significantly stimulated by ethanol supplementation (Supplementary Table S1).

Refeeding of CO led to increased alcohol production, particularly that of butanol and hexanol. Supplementation with ethanol further enhanced butanol and hexanol production. Notably, hexanol productivity over 144 h, during which supplemented ethanol was consumed, was much higher than that without ethanol supplementation (0.18 vs 0.077 g/L/day). Butanol productivity also increased by 2.0-fold (0.12 vs 0.058 g/L/day) over 144 h. Overall hexanol productivity during 312 h of cultivation was 0.18 and 0.14 g/L/day with and without ethanol supplementation, respectively. Further studies of the redirection of metabolism upon ethanol consumption are needed to understand the effect of ethanol supplementation on butanol and hexanol production.

### Comparison of This Study and Other Previous Studies

The culture conditions and medium compositions required to produce high alcohol content using *C. carboxidivorans* P7 via gas fermentation have been widely examined (Table 2). Phillips et al. (2015) investigated the effect of medium composition (specifically the trace metal composition) and culture techniques on the production of C4–C6 alcohols and reported 0.94 g/L of hexanol production. Ramió-Pujol et al. (2015) demonstrated that gas fermentation of *C. carboxidivorans* P7 at 25°C rather than 37°C was effective for producing alcohol by preventing acid crash, resulting in 0.84 g/L of hexanol production. The positive effect of low temperature on hexanol production was similar to that shown in Figure 1. Zhang et al. (2016) evaluated the effect of end products on the fermentation profiles at two temperatures, 25 and 37°C. Zhang et al. (2016) also achieved 2.32 g/L hexanoate production with 8 g/L butyrate supplementation at 25°C and 6.76 g/L ethanol production at 37°C with 2 g/L acetate; however, the hexanol concentration was not evaluated. According to Han et al. (2020), a decreased concentration of molybdenum in the medium shifted metabolism towards alcohol production. However, the main product was ethanol at 3.2 g/L, and hexanol production was only 0.6 g/L. Cheng et al. (2019) attempted to improve alcohol production by overexpressing *adh2*, which codes for aldehyde/alcohol dehydrogenase in *C. acetobutylicum*, and obtained 3 g/L ethanol and 0.27 g/L butanol; hexanol production was not monitored. Using a continuous gas-fed bioreactor, Fernández-Naveira et al. (2016) obtained 5.55 g/L of ethanol and 2.66 g/L of butanol; however, hexanol was not produced. Rückel et al. (2021) reported the production of hexanol using a fermenter but the concentration was only 0.16 g/L. Shen et al. (2017) optimized the composition of trace metals in the medium to enhance alcohol production and applied a timed control incubation temperature (37°C for 0–24 h, 25°C for 24–144 h), yielding a total alcohol titer of 6.97 with 1.67 g/L butanol and 1.33 g/L hexanol. This was the highest alcohol titer obtained for *C. carboxidivorans* P7 via gas fermentation, but ethanol was the main alcohol produced (3.97 g/L).

**TABLE 2:** Comparison of hexanol production performance obtained using *C. carboxidivorans* P7 with previous reports.

Type	Syngas supply				rpm	Temp (°C)	Working volume	Products (g/L)^a^						Reference
	CO	CO_2_	H_2_	kPa or mL/min				EtOH	BuOH	HeOH	AA	BA	HA	
Bottle	70	10	20	307 kPa	0	37	30 ml (in 282 ml bottle)	2.05	1.09	0.94	0.73	0.32	0.36	Phillips et al. (2015)
Bottle	32	8	32	100 kPa	100	25	20 ml (in 125 ml bottle)	1.48	1.07	0.84	1.65	-	1.05	Ramió-Pujol et al. (2015)
Bottle	56	20	9	200 kPa	100	37/25	30 ml (in 125 ml bottle)	3.97	1.67	1.33	(0.2)^b^	(0.1)^b^	(0.1)^b^	Shen et al. (2017)
Bottle	40	20	20	101 kPa	150	37	50 ml (in 160 ml bottle)	3	0.27	-	0.05	0.11	-	(Cheng et al., 2019)^c^
Bottle	50	35	15	200 kPa	200	37	20 ml (in 120 ml bottle)	1.66	0.84	0.3	0.15	0.05	-	Han et al. (2020)
Bioreactor	50	35	15	400 ml/min	200	37	3 L (in 7 L bioreactor)	3.2	1	0.6	2	0.3	-	Han et al. (2020)
Bioreactor	100	-	-	10 ml/min	250	33	1.2 L (in 2 L bioreactor)	4.41	2.30	-	1.04	-	-	Fernández-Naveira et al. (2016)
Bioreactor	4.0 L/h CO (pCO = 800 mbar) and 1.0 L/h CO_2_ (pCO_2_ = 200 mbar)					37	1 L (in 2.4 L bioreactor)	1.17	0.56	0.16	0.96	0.21	0.08	Rückel et al. (2021)
Bottle	70	-	-	100 kPa	100	30	20 ml (in 157 ml bottle)	1.21	1.21	1.90	0.73	0.44	0.59	This study
Bottle (ethanol addition)	70	-	-	100 kPa	100	30	20 ml (in 157 ml bottle)	−0.45^d^	1.60	2.34	0.82	0.38	0.41	This study

a EtOH, ethanol; BuOH, butanol; HeOH, hexanol; AA, acetic acid; BA, butyric acid; HA, hexanoic acid.

b Approximate values from graphs.

c *adh2* overexpression.

d Negative value indicates a decrease in ethanol concentration. Ethanol concentration was decreased from 2.40 to 1.01 g/L during 144 h of fermentation and then increased to 1.95 g/L after 312 h of fermentation.

As described above, the metabolite profiles of acetogens produced through syngas fermentation significantly depend on the fermentation conditions and media composition. However, the production of high-carbon alcohols, particularly hexanol, is limited. We achieved 1.90 g/L of hexanol production by refeeding 70% CO and cultivation at 30°C. To the best of our knowledge, this is the highest level of hexanol produced via gas fermentation reported to date and the first report of hexanol as the main product. Moreover, hexanol production was further improved to 2.34 g/L by ethanol supplementation. This notable result could be obtained by recognizing the effect of ethanol reassimilation on hexanol production during the gas fermentation of *C. carboxidivorans* P7. Considering that ethanol can be generated through acetogen fermentation (Lagoa-Costa et al., 2017) and the popular lignocellulosic bioethanol fermentation (Hoang Nguyen Tran et al., 2020), supplementing bio-ethanol could strengthen the potential of hexanol production via gas fermentation by not only enhancing the hexanol titer but also providing sustainable and effective ways for hexanol production. Additionally, utilizing ethanol can increase butanol production via gas fermentation, as illustrated in Figures 2 and 3. Further studies are required to achieve high and selective production of hexanol by genetically engineering *C. carboxidivorans* P7 as well as by removing hexanol during gas fermentation to prevent hexanol from inhibiting cell growth.

In conclusion, hexanol produced via gas fermentation using *C. carboxidivorans* P7 reached a concentration of 1.90 g/L, which is the highest titer reported to date and the first report of hexanol as the main product rather than C2–C4 metabolites. Favorable conditions for hexanol synthesis were incubation at 30°C and refeeding 70% CO gas. Hexanol production was further enhanced to 2.34 g/L by supplementation with ethanol, which facilitated chain elongation and alcohol production. This notable result was accomplished by optimizing culture conditions and by providing sufficient reducing equivalents through refeeding CO-rich gas and ethanol supplementation.

## Data Availability

The original contributions presented in the study are included in the article/Supplementary Material, further inquiries can be directed to the corresponding author.
