# Supplementary material for: Production of Hexanol as the Main Product Through Syngas Fermentation by Clostridium carboxidivorans P7
Source: Front Bioeng Biotechnol. 2022 Apr 25;10:850370. doi: 10.3389/fbioe.2022.850370 (PMC9081523; doi:10.3389/fbioe.2022.850370)
Supplement: Supplementary file 1 [file DataSheet1.docx]

**Supplementary Material**

Production of hexanol as the main product through syngas fermentation by *Clostridium carboxidivorans* P7

Hyun Ju Oh^1^, Ja Kyong Ko^1, 2^, Gyeongtaek Gong^1, 2^, Sun-Mi Lee^1, 2^,Youngsoon Um^1, 2^, *

^1^Clean Energy Research Center, Korea Institute of Science and Technology, Seongbuk-gu, Seoul, Republic of Korea.

Division of Energy and Environment

Technology, KIST School, University of Korea

Science and Technology (UST), Daejeon

Republic of Korea

^2^Division of Energy and Environment Technology, KIST School, University of Science and Technology (UST), Daejeon, Republic of Korea

**Figure S1**

| (A) | (B) |
| --- | --- |
|  |  |
|  |  |

**Figure S1.** (A) Cell growth (OD at 600 nm) and pH at 30°C, 33°C, and 37°C. (B) Ratio of C2, C4, and C6 (wt%) after fermentation at 30°C, 33°C, and 37°C.

**Figure S2**

| (A) | (B) |
| --- | --- |
|  |  |
| (C) |  |
|  |  |

**Figure S2.** Effect of initial CO ratio on (A) cell growth (OD at 600 nm) and pH, (B) CO gas consumption, and (C) hexanol production at 216 h. The initial headspace pressure was 150 kPa, and the syngas composition was CO:Ar = 30:70, 50:50, and 70:30. HeOH, hexanol

**Figure S3**

| (A) OD | (B) pH |
| --- | --- |
|  |  |

**Figure S3.** (A) Cell growth (OD at 600 nm) and (B) pH profiles during 70% CO batch fermentation with and without supplemented C2 compounds. EtOH, ethanol; AA, acetic acid

**Figure S4**

Supplemented ethanol

2NADH

Acetate

ATP

Acetyl-CoA

CO, CO_2_ , H_2_

Butyryl-CoA

Butyric acid

Butanol

Hexanoyl-CoA

Hexanol

2NADH

Ethanol

Hexanoic acid

Wood-Ljungdahl pathway

ATP

ATP

2NADH

2NADH

2NADH

2NADH

2 C_2_H_5_OH → C_4_H_9_OH + H_2_O

3 C_2_H_5_OH → C_6_H_13_OH + 2H_2_O

12 CO +5H_2_O → C_4_H_9_OH + 8CO_2_

18 CO +7H_2_O → C_6_H_13_OH + 12CO_2_

**Figure S4.** Hexanol synthesis pathway from syngas fermentation. Because acetyl-CoA and reducing equivalents are highly demanded for both chain elongation and alcohol production, the conversion of supplemented ethanol to acetyl-CoA and reducing equivalents may promote the production of butanol and hexanol.

**Figure S5**

**Figure S5.** Cell growth (OD at 600 nm) and pH profiles during 70% CO-refeeding fermentation with and without ethanol (2 g/L) supplementation

**Table S1**

**Table S1.** Summary of the ratios of (C4–C6 alcohols) to (C4–C6 acids) and C6 compounds to C4 compounds

|  | Fermentation conditions | C4–C6 alcohol to C4–C6 acid (g/g) | C6 compounds to C4 compounds (g/g) |
| --- | --- | --- | --- |
| For the section  “Effect of supplemented C2 compounds on hexanol production” | Control | 0.11 | 1.28 |
|  | Adding acetate | 0.05 | 0.81 |
|  | Adding ethanol | 0.56 | 1.33 |
|  | Adding acetate+ethanol | 0.33 | 0.82 |
| For the section  “Effect of syngas refeeding and supplementing ethanol on hexanol production” | Syngas refeeding | 2.99 | 1.51 |
|  | Adding ethanol  and syngas refeeding | 4.99 | 1.39 |
